# Commissioning and comprehensive evaluation of the ArcCHECK cylindrical diode array for VMAT pretreatment delivery QA

**DOI:** 10.1120/jacmp.v15i4.4832

**Published:** 2014-07-08

**Authors:** Vibha Chaswal, Michael Weldon, Nilendu Gupta, Arnab Chakravarti, Yi Rong

**Affiliations:** ^1^ Department of Radiation Oncology Ohio State University Columbus OH USA

**Keywords:** volumetric‐modulated arc therapy, ArcCHECK diode array, quality assurance, angular dependence, ArcCHECK commissioning, ArcCHECK evaluation

## Abstract

We present commissioning and comprehensive evaluation for ArcCHECK as a QA equipment for volumetric‐modulated arc therapy (VMAT), using the 6 MV photon beam with and without the flattening filter, and the SNC patient software (version 6.2). In addition to commissioning involving absolute dose calibration, array calibration, and PMMA density verification, ArcCHECK was evaluated for its response dependency on linac dose rate, instantaneous dose rate, radiation field size, beam angle, and couch insertion. Scatter dose characterization, consistency and symmetry of response, and dosimetry accuracy evaluation for fixed aperture arcs and clinical VMAT patient plans were also investigated. All the evaluation tests were performed with the central plug inserted and the homogeneous PMMA density value. Results of gamma analysis demonstrated an overall agreement between ArcCHECK‐measured and TPS‐calculated reference doses. The diode based field size dependency was found to be within 0.5% of the reference. The dose rate‐based dependency was well within 1% of the TPS reference, and the angular dependency was found to be ± 3% of the reference, as tested for BEV angles, for both beams. Dosimetry of fixed arcs, using both narrow and wide field widths, resulted in clinically acceptable global gamma passing rates on the 3%/3 mm level and 10% threshold. Dosimetry of narrow arcs showed an improvement over published literature. The clinical VMAT cases demonstrated high level of dosimetry accuracy in gamma passing rates.

PACS numbers: 87.56.Fc, 87.55.kh, 87.55.Qr

## I. INTRODUCTION

Intensity‐modulated radiation therapy (IMRT) has become a widely accepted and efficient treatment technique for many types of cancers. With its highly modulated conformal beams it has the advantage of selective dose escalation to the irregularly shaped tumor sites, while effectively avoiding the adjacent normal tissue and organs at risk. Volumetric‐modulated arc therapy (VMAT) combines the benefit of dose conformity and efficiency of arc delivery by treating patients with the highest level of beam orientations in the shortest possible time. VMAT delivery system provides continuous variation of dose rate, gantry speed, and multileaf collimator (MLC) leaf position.^1^, ^2^ While the gantry is rotating, beam aperture is changed continuously with dose rate and gantry speed varied.

Very recently, investigators have shown that gamma analysis‐based passing rates in a composite plan, as well as single field planar dosimetry of highly modulated IMRT fields, are insensitive to important dosimetric inaccuracies of the plan, and thus potentially mask clinically relevant errors.^3^, ^4^, ^5^, ^6^, ^7^ Such studies, and VMAT's complex dose delivery, mandate development of a comprehensive patient QA, preferably based on 3D dose delivery and assessment of dose inside the patient, rather than in a planar phantom. Establishing patient‐based QA practice requires a whole new repertoire of a 3D dose phantom and auxiliary software with capability to reconstruct measured QA phantom dose onto patient's anatomy. Manufacturers of QA equipments have come up with various phantoms/QA devices to assist the commissioning of VMAT and the related patient‐based pretreatment QA. Some of these are: ${\text{Delta}}^{4}$ diode array phantom (ScandiDos Inc, Uppsala, Sweden), ArcCHECK cylindrical diode array and 3DVH (Sun Nuclear Inc, Melbourne, FL), COMPASS system (IBA Dosimetry, Schwarzenbruck, Germany), and Dosimetry Check (Math Resolutions LLC, Columbia, MD).

In this study, we will report comprehensive commissioning and evaluation tests conducted on the ArcCHECK 3D diode array. Investigations into the use of its auxiliary softwares, control point analysis, and 3DVH for VMAT pretreatment customized patient QA are underway and will be published in a separate manuscript.

Proper commissioning and evaluation of a QA device are the most critical steps in building the confidence, minimizing the error, and understanding the variations and responses in dose evaluation. ArcCHECK is a cylindrical acrylic phantom with a three‐dimensional array of 1386 diode detectors with 10 mm spacing. The active detector size is 0.8 mm×0.8 mm. The detectors are placed in a spiral geometry across the length of the 21 cm diameter cylinder, in a non‐overlapping beam's eye view (BEV) geometry, to reduce the shadowing and increase the effective detector density in BEV. There is a 15 cm diameter cavity in the center of the phantom that can hold a plug for filling in the cavity for continuous geometry. There is also an auxiliary insert with a cavity for ion chamber placement for absolute dose measurement at the center of the phantom. ArcCHECK measures radiation in real time with 50 ms update rate, saves all measurement data as a function of time, and makes both relative and absolute dose measurements.

Prior independent* evaluation studies on ArcCHECK reported on the angular and directional dependencies and field size dependencies,^8^, ^9^ and its limitation for dosimetry of fixed arcs.^(8)^ Most recently, Li et al.^(10)^ reported detailed evaluation of ArcCHECK and minor directional dependencies with minimal clinical impact on VMAT plans. The manufacturer has upgraded the ArcCHECK software and hardware since 2011. After the software was renamed as SNC Patient in May 2011 (SNC Patient, version 6.0), upgrades have been made for enhanced features and superior dose corrections relevant to ArcCHECK measurements. These include angular dependence correction with virtual inclinometer, field size dependence correction, inhomogeneity correction, and measurement merge for large fields. The device has been upgraded, as well, to better accommodate the flattening filter‐free (FFF) beams for all clinically available linacs and energies via a new measurement circuit enhancement for high dose‐per‐pulse beams (Sun Nuclear Application Note 02‐11: ArcCHECK for Flattening Filter Free Linac Beams, Rev A).

In this study, we commissioned the ArcCHECK device under a strict comprehensive testing procedure, especially in consideration with the previous finds and upgrades, and investigated its usefulness (and limits) for patient‐specific VMAT QA in our clinic.

## II. MATERIALS AND METHODS

All measurements were done using the TrueBeam STx accelerator (Console version 1.6; Varian Medical Systems, Palo Alto, CA) with a 6 MV beam with and without flattening filter (denoted as 6X and 6F beam). Varian Eclipse treatment planning system (TPS) and analytical anisotropic algorithm (AAA) were used (version 10.0.39) for calculating reference dose grids. All planned doses in this study were calculated using a symmetric 3D dose grid size of 2 mm×2 mm×2 mm. The angular resolution is set to 4° for conformal arcs and defaulted to every control point for VMAT, representing the clinically acceptable criterion currently in use at our clinic. All the dose measurements were performed using ArcCHECK with the center plug inserted. Measurements recorded by ArcCHECK phantom had correction factors for background radiation, angular dependence, field size dependence, and heterogeneity correction automatically applied prior to saving the measured dose file. All comparisons performed in this study have the above‐mentioned correction factors applied. All of these correction factors are determined at the manufacturer's site during initial calibration of ArcCHECK and provided as text files with the SNC software. Post‐irradiation, when the “Stop” button is clicked at the end of a measurement, the software displays progress bars as it analyzes each update, calculates each gantry angle, and applies all corrections (background correction, angular correction, heterogeneity correction, field size correction). The SNC software applies the field size correction factors by calculating field size and applying the appropriate correction factor according to the energy, diode location, and plug usage. The angular correction is applied using: a Monte Carlo calculation‐based average angular dependence factor that accounts for the internal structure of the diode which is not invariant to the beam; another factor called ‘Individual deviation’, representing the deviation of the detector's angular dependence with respect to the average angular dependence; and the virtual inclinometer that reconstructs gantry angle based on spatial dose distribution in ArcCHECK every 50 ms, for the application of angular dependence factor and individual deviation factor. The reader is guided to the SNC user manual for detailed explanation of the measurement of correction factors.

TPS‐calculated dose was used as the reference for the ArcCHECK evaluation testing. This is a reasonable approach knowing that the AAA algorithm is benchmarked within a dose accuracy of 2 mm/2% for both 6X and 6F beams, comparing to measurements and Monte Carlo simulations.^11^, ^12^, ^13^, ^14^, ^15^, ^16^, ^17^, ^18^ Our study focused on evaluating ArcCHECK against a fully commissioned in‐use TPS, aiming to understand its performance and deviations with respect to the TPS as a VMAT QA tool. The comparison of phantom‐measured versus TPS‐calculated dose was based on profiles and 3D gamma analysis.^19^, ^20^ For dose difference tolerance of D% and a distance‐to‐agreement (DTA) tolerance of R mm, the gamma passing rate is abbreviated as “γ D%/Rmm” in the proceeding document. The global and local gamma indices (γ index) were both computed for 3 mm/3% and 2 mm/2% criterion using the SNC software. Gamma evaluations were performed in the absolute dose mode, with the default normalization to the maximum dose in the curved plane and a low‐dose threshold of 10%, to restrict the analysis to the clinically relevant areas. The global gamma analysis involves the Van Dyk % difference,^(21)^ which is the percent difference between any measured point and the corresponding plan point normalized to a common point (usually maximum dose point). The local gamma analysis involves the points passing within the set criteria when co‐located measurements and calculated points are compared.

### A. Commissioning for the ArcCHECK diode array phantom

#### A.1 Absolute dose calibration and array calibration

The absolute dose was calibrated against the ion chamber measurement in a slab Solid Water phantom, following the manufacturer recommendation. The array calibration was performed following the twelve‐step procedure instructed by the SNC Patient software.

#### A.2 PMMA density determination

For TPS dose calculation, the manufacturer recommends considering ArcCHECK as a homogeneous phantom with an assigned density close to PMMA material, as the phantom heterogeneity is accounted for in the correction factors. A bulk density value best suitable to the TPS was determined and assigned. The PMMA density verification resulted in assignment of a mass density of $1.19\;g/{\text{cm}}^{3}$ (CT value 282) to the virtual phantom in the TPS for all dose calculations using ArcCHECK. This is a very important step in commissioning since it models the ArcCHECK in the TPS; systematic errors, if any, will get carried over in all the future dose calculations. Therefore, to ensure minimal systematic error progression in a QA process, the bulk density correction determination and verification should be done correctly, prior to any characterization/evaluation testing. Recent publication by Nelms et al.^(7)^ has reported systematic error as high as 8% due to incorrect density assignment and also their masking due to lose tolerances of 3%/3 mm global gamma analysis used clinically for VMAT QA.

#### A.3 Constancy of device and setup accuracy

ArcCHECK response constancy was checked using $10\times 10\;{\text{cm}}^{2}$ static fields at 0°, 90°, 270°, and $10\times 10\;{\text{cm}}^{2}$ arc fields after the device was set up. This continuing baseline‐check procedure at our institute has two‐fold benefit of a) dosimetric setup accuracy testing and b) device constancy measurements. Over a time period spanning four months, constancy measurements were gathered on five different instances, before starting the measurements. Gamma passing rates, as well as the mean and standard deviation in dose measurements, were reported. Measurement at gantry angle 180° was omitted, since it involved the couch correction factor and was evaluated in depth in the Materials and Methods section B.7.

### B. Evaluation testing for the ArcCHECK diode array phantom

Following absolute dose calibration and PMMA density verification, ArcCHECK was evaluated for its response dependency on linac dose rate, instantaneous dose rate, field size, beam angle, couch insertion, and scatter dose. The stability and consistency of response were also studied. Dosimetry accuracy evaluation involved fixed arcs and VMAT patient plans.

#### B.1 Dose‐rate (pulse rate) dependence

**Figure 1:**
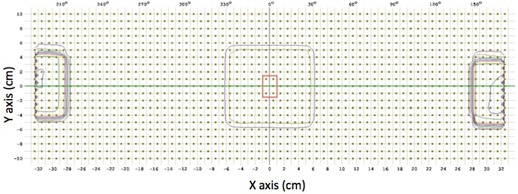
Location of six diodes within 5 mm of the central axes of phantom used for analysis of dose‐rate dependency, dose‐per‐pulse response, and field‐size dependence test. Diodes located inside the red rectangle around origin are the analysis diodes; these lie at 5 mm from CAX.

Measurements were made with a $10\times 10\;{\text{cm}}^{2}$ field size, 100 cm SAD geometry for both 6X and 6F energies. The dose‐rate dependence of ArcCHECK was evaluated for the dose rates of 20, 40, 200, 400, 600 MU/min for 6X and 600, 800, 1000, 1200, and 1400 MU/min for 6F. Average measured dose from the six diodes within 5 mm of the central axes of the ArcCHECK cylinder were used for analysis. If the phantom is visualized as a cut along a transverse cross section from the bottom and spread in 2D plane, all diodes get spread on the same plane, then the diodes used for measurement are located within the radius of 5 mm from the CAX around isocenter, and no diode is directly present on the central axis (CAX) of the radiation beam. Figure 1 shows the location of these six diodes. Averaging the doses within 5 mm central region of the field is considered adequate information for dose‐rate dependency analysis.

#### B.2 Dose‐per‐pulse (instantaneous dose rate) dependence (SAD dependence)

Measurements were made with a $10\times 10\;{\text{cm}}^{2}$ field size, on a varying SAD geometry (90, 100, 110, and 120 cm), 100 MU for both 6X and 6F energies. This test examined the instantaneous dose‐rate dependence of ArcCHECK diodes resulting from random variations in recombination‐generation centers due to changing proximity to the radiation source. Measured dose for analysis was computed in the same manner as the average dose from the 6 central diodes in the isocentric plane along both the axes (Materials & Methods section B. 1). Dose‐per‐pulse dependence was obtained after factoring out the inverse square factor from the readings.

#### B.3 Field size dependence

Field size dependence of diodes was evaluated using 100 cm SAD geometry and 100 MU delivery for four static field sizes (5×5, 10×10, 20×20, and $5\times 5\;{\text{cm}}^{2}$). Dose measurements for analysis were taken from six central diodes located within 5 mm of the isocenter (Materials & Methods section B.1).

#### B.4 Angular dependence

**Figure 2:**
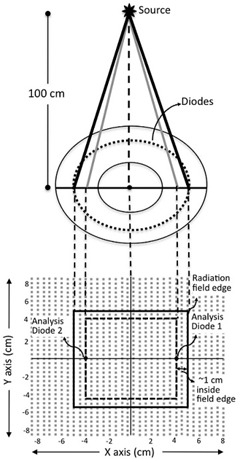
Measurement analysis geometry of the angular dependence test. Upper part of the figure depicts the measurement geometry of a $10\times 20\;{\text{cm}}^{2}$ field irradiation on a central axial cut of ArcCHECK. Lower part depicts analysis geometry: 2D projections of the radiation field edge and ~ 1 cm inside are shown on the 2D plane containing all the diodes. Average measured dose from 2 “analysis diodes” is used as one data point for angular dependence.

The angular response of ArcCHECK diodes was derived from the same data that were used for the field size dependence. For this, average dose response of diodes (either side of the central axis) that lay in the path of the most divergent rays was considered. Ideally these would be the field‐edge diodes. However, since the TPS algorithms, as well as the measurement devices, have a limited accuracy of penumbra beam modeling/measurement, the penumbra dose values from the TPS have a higher uncertainty associated with them. Considering this, we measured the angular dependence using diodes that lay about 1 cm inside the outer geometric field edge in the central axial plane. Figure 2 shows the schematic measurement‐analysis geometry depicting the location of two diodes used for angular dependence analysis. Data from eight diodes (two diodes in line with most divergent rays, per square field size, over four square field sizes) were used for getting the angular response of diodes with respect to the range of angles that fall in the beam's eye view (BEV) alone. The angle of incidence of the divergent beam on these diodes with respect to the central axis of the incident radiation beam ranges between 0.86° to nearly 6°, for the field sizes ranging between $5\times 5\;{\text{cm}}^{2}$ and $20\times 20\;{\text{cm}}^{2}$.

#### B.5 Symmetry of response

Irradiations using wide open arcs were used for the classic phantom flip test for checking the relative dose response of the dosimeter phantom. A wide open field fixed arc beam was measured, and measurement was repeated after rotating the phantom by 180° along the longitudinal axis. Wide field sizes ensured inclusion of all detectors in high/low dose gradient areas. Two wide field arcs of field sizes $10\times 15\;{\text{cm}}^{2}$ and $25\times 25\;{\text{cm}}^{2}$, each spanning 358°, were used in the detector flip test; 400 MU/arc were delivered while phantom in SAD setup.

#### B.6 Scatter response

Scattered radiation‐based overresponse of diodes was measured for varying amount of scatter derived from irradiations of field sizes of 5×5, 10×10, 15×15, and $20\times 20\;{\text{cm}}^{2}$. Measurements were made at distances varying between 1 cm to 8 cm (depending on geometric limitations of the phantom) from the field edge, in both axial and coronal directions.

#### B.7 Effect of couch insertion in TPS

Measurements taken at the gantry angle 180° using a $10\times 10\;{\text{cm}}^{2}$ field were compared with the dose calculated in the TPS with and without the couch insertion. Since the beam travels through the carbon fiber couch (BrainLAB couch top, BrainLAB, Inc, Westchester, IL) with distinct variable relative electron densities (RED) (couch interior: RED $0.1179\;g/{\text{cm}}^{3}$, couch surface: RED $0.7000\;g/{\text{cm}}^{3}$), it offers considerable attenuation to the photons. This comparison is important to set a protocol for clinical practice and QA procedures.

#### B.8 Dosimetry of full arcs with fixed apertures

Dosimetry accuracy in arc mode delivery is the next most logical test. Arcs with varying fixed apertures ranging from narrow aperture to wide open apertures were used for dosimetric evaluation. Reference dose datasets using arc apertures of 2×10, 3×10, 5×10, 5×5, 10×10, 5×25, 10×25, and $25\times 25\;{\text{cm}}^{2}$ were calculated in the TPS with a symmetric 3D dose grid resolution of 2 mm and 4° control point angle (CPA) increment. Gamma passing rates were computed for verification of dosimetric accuracy.

#### B.9 Dosimetry of VMAT plans

After verifying phantom's performance in the conformal arc mode, the dosimetric evaluation of Eclipse RapidArc plans was performed. Five RapidArc plans (three clinical plans and two RPC phantom treatment plans) were used for evaluating phantom's dosimetric accuracy. Plans involved treatment sites: brain, RPC‐spine, RPC‐head and neck, scalp, and a whole brain retreatment with hippocampal sparing (RTOG 0933). Verification plans were calculated on the homogeneous virtual ArcCHECK with the PMMA density overridden, using a symmetric 2 mm 3D dose grid and every control point increment. Couch top was included into the calculation as an ROI. The plans involved two, three or four noncoplanar arcs with stringent dose constraints for normal tissue, organs, and risk sparing. For the purpose of VMAT QA in this paper, all plans were calculated on the phantom with couch rotation set to zero, and measurements were taken in the same geometry.

## III. RESULTS & DISCUSSION

The analysis of all the tests conducted was done either by analyzing the trend of absolute dose variation under certain irradiation conditions or/and geometries as measured by diodes, or by comparison of the measured dose with the Eclipse TPS‐calculated dose using gamma analyses. Results are listed in the same order as in the Materials & Methods section, so the reader can coordinate the perusal easily.

### A. Constancy of device, setup accuracy

The global gamma passing rates at 2%/2 mm level of the $10\times 10\;{\text{cm}}^{2}$ field irradiations at 0°, 90°, 270°, and a full arc rotation, over a time period spanning nearly four months, are above 99%.

### B. Dose‐rate (pulse rate) dependence

The average absolute dose measured over six central diodes within 5 mm of the central axis of ArcCHECK did not vary by more than 0.9% for the 6X beam and 0.4% for the 6F beam, over different dose rates. The standard deviation from average measured dose was under 0.4 cGy in the central 5 mm region. The dose output measured by ArcCHECK diodes was stable (within 1%) over the whole range of dose rates (20‐600 MU/min for 6X and 600‐1400MU/min for 6F beam).

### C. Dose‐per‐pulse (instantaneous dose rate) dependence

**Figure 3:**
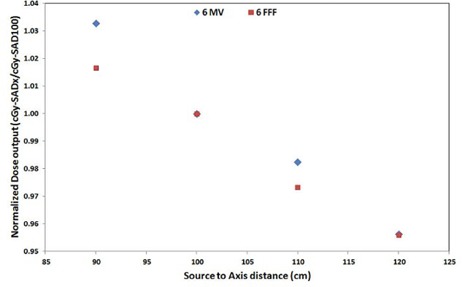
Dose‐per‐pulse (instantaneous dose rate) dependence of 6X and 6F beam as measured by the average dose over six central diodes at 5 mm from the isocenter in axial and transverse directions. Dose is normalized to the dose at 100 SAD.

Dose‐per‐pulse dependence was demonstrated as an over‐response of diodes seen in closer proximity to the source and an under‐response farther from the source. The dose‐per‐pulse dependence of ArcCHECK after factoring out the inverse‐square dependence of dose was plotted in Fig. 3 and data tabulated in Table 1. For 6X beam, when ArcCHECK was placed closer to the beam than 100 cm SAD, diodes overresponded by 0.41%/cm as calculated at a distance of 90 cm from the source; and farther from the beam they under‐responded by 0.15%/cm as calculated at a distance of 120 cm from the source. For 6F beam, this over‐response closer to the linac source was estimated to be 0.21%/cm; and the under‐response farther from the source was 0.15%/cm.

**Table 1:** Dose‐per‐pulse (instantaneous dose rate) dependence of central ArcCHECK diodes

*SAD (cm)*	*Average Absolute Dose*	*ISF*	*Normalized Output*	*Dose/pulse (DPP)*	*% Difference*	% Diff/cm
*6 MV*						
90	155.59	1.23	1.28	1.03	4.1	0.41
100	122.02	1.00	1.00	1.00	0.0	0.00
110	99.07	0.83	0.81	0.98	−1.5	−0.15
120	81.04	0.69	0.66	0.96	−3.0	−0.15
*6 FFF*						
90	152.16	1.23	1.26	1.02	2.1	0.21
100	121.23	1.00	1.00	1.00	0.0	0.00
110	97.52	0.83	0.80	0.97	−2.2	−0.22
120	80.49	0.69	0.66	0.96	−3.1	−0.15

Due to the change in indirect recombination rate, dose‐per‐pulse dependence is seen in addition to the change in dose rate, merely due to increasing or decreasing distance of the diode detector to the linac source. For both the beams, when the SAD was decreased to 90 cm, the response of the diode detector was nearly 4.0%(6X) and 2.1%(6X) higher, in addition to 23% (ISF=1.23) increase that is seen purely due to increased proximity to the radiation source. Similarly, when the SAD was increased beyond 100 cm to 110 cm and 120 cm, the response was dropped nearly 1.5% and 3.0% more than what it should have dropped to if there was no random variation due to instantaneous dose rate for the 6X beam; for the 6F beam, response dropped by 2.2% (110 cm SAD) and 3.1% (120 cm SAD).

### D. Field size dependence

With field size correction factors applied in the software, the remaining discrepancies between measured and calculated were within 0.4% for field sizes ranging from 5×5 to $20\times 20\;{\text{cm}}^{2}$. A composite gamma analysis demonstrated high gamma passing rates, as summarized in Table 2. The behavior of a diode detector with a changing field size is typical of any other radiation detector depicting increase in total scatter factor (predominantly phantom scatter, Sp), as the field size increases and decreases in the opposing case. High gamma passing rates resulting from diode‐measured and TPS‐calculated doses, as well as direct diode‐to‐TPS comparison, showed no special diode‐based field size dependence. This is considered a result of a field size correction factor that gets automatically applied to the measured dose when saved using the SNC Patient software version 6.2. These correction factors are with respect to gantry's angular positions, and range between field sizes $2\times 2\;{\text{cm}}^{2}$ and $25\times 25\;{\text{cm}}^{2}$ for each energy.

**Table 2:** Average gamma passing rates for different square field sizes varying from $5\times 5\;{\text{cm}}^{2}$ to $20\times 20\;{\text{cm}}^{2}$

*Static Field Sizes*	γ (3%/3 mm)≤1		γ (2%/2 mm)≤1	
*(cm^2^)*	*Global*	*Local*	*Global*	*Local*
*6 MV*				
5×5	100	89.4	99.7	81.8
10×10	100	97.4	100	94.4
15×15	99.9	95.0	98.9	85.7
20×20	99.6	86.7	93.9	74.7
*6 FFF*				
5×5	100	98.5	100	90.9
10×10	100	94.3	100	90.0
15×15	100	85.7	98.8	99.3
20×20	95.6	76.9	90.9	62.4

### E. Angular dependence

The angular response of ArcCHECK diodes was derived from field size data by using the dose measured by diodes that lay in the path of the most divergent rays (∼1 cm infield the geometric field edge). The angles that these diodes make with incident beam lie between the range of 0.86° to nearly 6°. This range covers the clinically relevant angular incidence range of entry diodes that are always perpendicular to the beam at zero gantry and couch angles.

**Figure 4:**
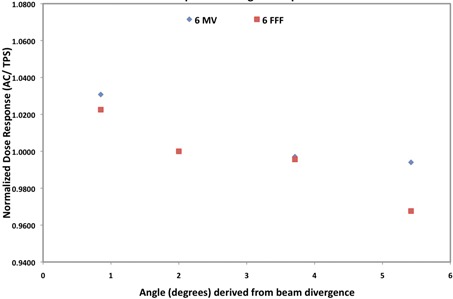
Angular dependence factors of ArcCHECK diodes normalized to the diode reading with the $10\times 10\;{\text{cm}}^{2}$ field size.

Figure 4 shows the angular dependence of ArcCHECK diodes with respect to the TPS normalized to the diode reading at $10\times 10\;{\text{cm}}^{2}$ field size. For both 6X and 6F, angular dependence factor of the ArcCHECK diodes decreased as the beam divergence increased. With respect to the TPS, the ArcCheck measured doses were up to 1% and 3% lower for 6X and 6F, respectively, for the angular incidence derived from the $20\times 20\;{\text{cm}}^{2}$ field size beam, as compared to a fairly perpendicularly incident beam ($5\times 5\;{\text{cm}}^{2}$) for which the measured response is nearly 2.5% higher from the TPS for both energies. Some sources of uncertainty in this test are the proximity of the diodes to penumbra of the beam and TPS model uncertainties of dose calculation in this region. Authors' recommendation to the reader intending to repeat this evaluation test is to use one large flush field, and derive the data over several diodes from the internal 80% region of the field, to avoid these uncertainties.

### F. Symmetry of response

Symmetry of response was measured by comparing the measured dose with and without the flipped geometry with the reference dose. Table 3 summarizes the gamma passing rates for the two wide arcs delivered. Gamma analyses demonstrated high passing rates for both global and local comparisons, indicating a high symmetry of response.

**Table 3:** Global and local gamma passing rates of the measured arcs (two exposures with phantom rotated on its long axis) as compared with the arcs calculated in the TPS

*Arcs (cm^2^)*	γ (3%/3 mm)≤1		γ (2%/2 mm)≤1	
	*Global*	*Local*	*Global*	*Local*
10×25 arc	99.9%	99.9%	98.3%	98.1%
10×25 arc flip	99.8%	99.8%	97.6%	97.5%
25×25 arc	99.8%	99.6%	95.6%	95.0%
25×25 arc flip	99.6%	99.4%	94.5%	94.0%
Average	99.8%	99.7%	96.5%	96.2%
Std Dev	0.13	0.22	1.76	1.96

### G. Characterization of scatter response

For 6X beam, the scatter values, both axial and coronal, measured at 1 cm to 8 cm out of field, were on an average of 30% to 10% higher than the TPS. For 6F beam, the scatter values were even higher, on an average of 50% to 15% higher than than TPS, measured at 1 cm to 8 cm out of field. This can be explained by the diodes' energy dependence to low‐energy photons; thus an over‐response is expected. However, literatures have shown that the Eclipse TPS underestimated out‐of‐field doses^22^, ^23^ by an average of 40% over the range of distance from 3.75 to 11.25 cm from the field edge.^(22)^ Therefore, the over‐response from the diodes measurement might not be as dramatic, compared to the actual dose delivered. The level of these scatter doses are well below the 10% threshold used in gamma calculation and should not affect the clinical QA analysis.

### H. Effect of couch ROI

The global gamma passing rates showed a clear improvement in passing rates when couch ROI was included in TPS dose calculations. γ (3%/3 mm) increased from 89.5 to 100, and γ (2%/2 mm) increased from 73.1 to 95.5 for a $10\times 10\;{\text{cm}}^{2}$ fixed arc irradiation. This is an experiment worth conducting once to remember the consequences of a missing couch when passing rates fail miserably. At our institution, adding an extra couch ROI is part of the protocol for using ArcCHECK for treatment QA. This is in accordance with a similar observation reported by Vanetti et al.,^(24)^ that there are significant discrepancies of potential clinical impact at the level of the target volumes if calculations are performed without the couch and delivery is performed with couch.

### I. Dosimetry of full arcs with fixed apertures

Whereas wide open arcs measure the dosimetric accuracy of uniformity of dose response in the center of cylinder of ArcCHECK phantom, narrow aperture arcs quantify the errors in the peripheral dose as an effect of small arc approximation in the TPS (discretization of continuous arcs into static beams in the TPS). Dosimetry results of full arcs with narrow open fixed aperture (field widths 5 cm and below) were tabulated in Table 4 as the global gamma passing rates. The calculations of arcs in the TPS rely heavily on the discretization of continuous arc into a number of static beams. The robustness of small arc approximation for rotational therapy (like VMAT or conformal arcs) diminishes as the radial distance of the point of interest increases away from the center.^(25)^ The impact of the unstable reference (calculated) dose away from the isocenter with respect to control‐point spacing in VMAT has been reported by Feygelman et al.^(8)^ for narrow arcs. Very poor gamma passing rates were reported in their study (as low as 25%). The newer version of SNC Patient software involves improvements of accuracy in the measured dose through a set of correction factors, as mentioned previously. Moreover, the previous versions of software only considered the planned (calculated) dose for the threshold dose, whereas the current version includes both the planned and the measured dose. This may result in inclusion of more points for the gamma analysis and, hence, the higher passing rates. However, for arcs as narrow as $2\times 10\;{\text{cm}}^{2}$, the clinically relevant gamma passing rate of 3%/3 mm gamma index is still low (75%), but it is a significant improvement from the literature.^(8)^ This effect on the VMAT plans with narrow apertures is further discussed in the next section.

Results of dosimetry of full arcs with wide open fixed aperture (field widths 5 cm and above) are tabulated in Table 5 as local and global gamma passing rates. All gamma analyses are done in absolute dose mode with 10% dose threshold. Except for γ (2%/2 mm) for the unfiltered beam (6 FFF) which is 87.97 for the global comparison and 85.5 for the local comparison, the average gamma passing rates are high (≥95%) everywhere else. This shows a very good agreement between ArcCHECK‐measured doses and TPS‐calculated doses. These results demonstrate an improvement in performance of ArcCHECK phantom, as compared to the poor passing rates reported by Feygelman et al.^(8)^ for fixed arcs, indicating superior performance after software upgrades in SNC Patient software version 6.2 used for all analysis in this paper. The lower γ (2%/2 mm) passing rates indicate higher sensitivity of this metric because of tighter tolerance involved in its calculation. The sensitivity of this metric was recently investigated by

**Table 4:** Dosimetry of narrow beam aperture arcs

	*6 MV*		*6 MV*		*6 FFF*		*6 FFF*	
*Arcs (cm^2^)*	γ (3%/3 mm)≤1		γ (2%/2 mm)≤1		γ (3%/3 mm)≤1		γ (2%/2 mm)≤1	
	*Global*	*Local*	*Global*	*Local*	*Global*	*Local*	*Global*	*Local*
*Narrow Width Arcs*								
2×10	75.0%	‐	59.2%	‐	89.9%	‐	69.9%	‐
3×10	87.9%	‐	52.5%	‐	96.1%	‐	68.1%	‐
5×5	96.1%	80.3%	75.0%	66.4%	99.9%	90.0%	88.2%	80.0%

**Table 5:** Dosimetry of full arcs with wide open apertures

	*6 MV*		*6 MV*		*6 FFF*		*6 FFF*	
*Arcs (cm^2^)*	γ (3%/3 mm)≤1		γ (2%/2 mm)≤1		γ (3%/3 mm)≤1		γ (2%/2 mm)≤1	
	*Global*	*Local*	*Global*	*Local*	*Local*	*Global*	*Local*	*Global*
*Wide Open Arcs*								
5×10	99.9%	77.2%	76.5%	63.9%	99.3%	84.3%	77.8%	74.8%
10×10	100%	81.7%	95.5%	75.5%	100.0%	99.9%	85.1%	84.0%
10×25	99.9%	99.9%	98.3%	98.1%	100.0%	99.9%	98.1%	97.0%
5×25	99.9%	99.9%	94.1%	93.6%	91.6%	88.5%	67.0%	63.6%
25×25	99.8%	99.6%	95.6%	95.0%	98.6%	95.9%	80.7%	74.0%
Average	99.9%	95.3%	95.9%	90.6%	99.5%	98.6%	88%	85.5%
SD	0.00	0.09	0.02	0.10	0.01	0.03	0.09	0.16

Nelms et al.^(7)^ in a paper discussing practical examples of masking of systematic errors by the global 3%/3 mm criterion. Their investigations indicated the higher sensitivity of the local γ(2%/2 mm) analysis towards TPS dose calculation‐based uncertainties involving beam model inaccuracies in the high gradient dose regions, underestimation of dose for narrow MLC segments, and even phantom misalignment in the TPS and incorrect density assignment.^(7)^

### J. Dosimetry of VMAT plans

For the analysis parameters we typically use in our clinic, the ArcCHECK demonstrated high average global gamma passing rates at γ (3%/3 mm) and γ (2%/2 mm) for the unfiltered 6F beam (98.9% and 95.2%, respectively), as tabulated in Table 6. For the 6X beam, the average global γ (2%/2 mm) was slightly lower than 90% (1.4% lower), whereas γ (3%/3 mm) was 96.06%. All the considered VMAT plans passed the clinically accepted QA pass criteria of γ (3%/3 mm)>90%, for IMRT and VMAT plan QA.

Of the two cases with low 2%/2 mm gamma passing rates, the VMAT plan for RPC‐spine case includes four varying aperture partial arcs with the maximum field width of $4.6\times 7.0\;{\text{cm}}^{2}$. As shown in the previous section, we expected to see low passing rate for narrow arcs, due to two main reasons — the instability of small arc approximation in dose calculation of the peripheral regions by the TPS, and the heavy dependence of ArcCHECK on peripheral diodes for dose measurements. However, for the studied cases, plans are considered a clinical pass, since the passing rate for γ (3%/3 mm) is >90% of the points under consideration.

**Table 6:** Gamma passing rates (global) of delivered VMAT plans versus planned (calculated) doses in the TPS

	*6 MV*		*6 FFF*	
	γ	γ	γ	γ
*VMAT Plan*	(3%/3 mm)	(2%/2 mm)	(3%/3 mm)	(2%/2 mm)
Brain retreatment (4 Arcs)	96.1%	91.0%	98.3%	93.3%
RTOG0933 (2 Arcs)	99.9%	97.4%	99.9%	98.3%
Scalp (3 Arcs)	98.5%	92.0%	99.4%	97.2%
RPC‐HN (2 Arcs)	94.7%	86.3%	99.0%	95.7%
RPC‐Spine (4 partial Arcs)	91.1%	76.6%	97.9%	91.6%
Average	96.1%	88.7%	98.9%	95.2%
SD	3.4	7.8	0.8	2.8

## IV. CONCLUSIONS

We presented the results of commissioning and comprehensive testing procedures we used to evaluate the cylindrical diode array phantom ArcCHECK for dosimetry of VMAT QA in our center, using the 6 MV photon beam with and without the flattening filter (designated as 6X and 6F, respectively). ArcCHECK diodes, when tested for their dependency on linac dose rate, dose per pulse, field size, and angular response of diodes, scatter response demonstrated a clinically acceptable response. The diode‐based field size dependency was found to be 0.5% within the reference TPS dose, which is considered a direct result of improved field size correction factors in the new SNC Patient software. The dose‐rate dependence was well within 1% for both beams. The instantaneous dose rate‐based diode response was evaluated. The angular dependency was found to be up to ±3% of the TPS reference, as tested over BEV angles only, for both beams.

Dosimetry of fixed arcs, using both narrow and wide field widths, were analyzed using both global and local gamma comparisons at the levels of 3%/3 mm and 2%/2 mm. The fixed arc dosimetry resulted in clinically acceptable gamma passing rates on 3%/3 mm level; however, at 2%/2 mm level, the passing rates were lower. Dosimetry of narrow arcs showed an improvement over published literature, presumably resulting from improvements in the software handling the data analysis, offsetting the overstated effect of peripheral placement of diodes in ArcCHECK. Dosimetry of clinical VMAT cases was analyzed using the global gamma analysis at 3%/3 mm and 2%/2 mm levels. The clinical VMAT cases demonstrated high level of dosimetry accuracy in global gamma passing rates at the levels of 3%/3 mm for both 6X and 6F beams. At the level of 2%/2 mm, two VMAT cases involving the narrow heavily modulated arcs demonstrated lower passing rates. After the commissioning and comprehensive evaluation testing of ArcCHECK, we consider phantom well‐suited for VMAT QA in our clinic.

In the absence of written recommendation on acceptable tolerance levels for commissioning and evaluation of ArcCHECK 3D diode array, our commissioning and evaluation study included gamma comparisons, both global and local, at the level of 2%/2 mm in almost all dosimetric and evaluation tests. This tighter tolerance level than the customarily acceptable global 3%/3 mm gamma analysis was chosen to gain confidence in device's performance. Tests were detailed, keeping in mind previous publications and new upgrades to the ArcCHECK hardware and software. Our tests demonstrate the impact of applications of the new correction factors and device upgrades on clinical measurement for VMAT QA, using 6 MV photon beam with and without the filter.

In light of recent publications and our own finds of higher sensitivity of 2%/2 mm gamma analysis in this paper, we intend to use it as one of the VMAT QA metrics for patient plan evaluations during pretreatment delivery QA. Investigations into the use of more directly clinically relevant and sensitive QA tools like 3DVH and CPA for VMAT QA are underway.

## Supporting information

Supplementary Material
