# Supplementary figures and images for: Commissioning and comprehensive evaluation of the ArcCHECK cylindrical diode array for VMAT pretreatment delivery QA
Source: J Appl Clin Med Phys. 2014 Jul 8;15(4):212–25. doi: 10.1120/jacmp.v15i4.4832 (PMC5875521; doi:10.1120/jacmp.v15i4.4832)

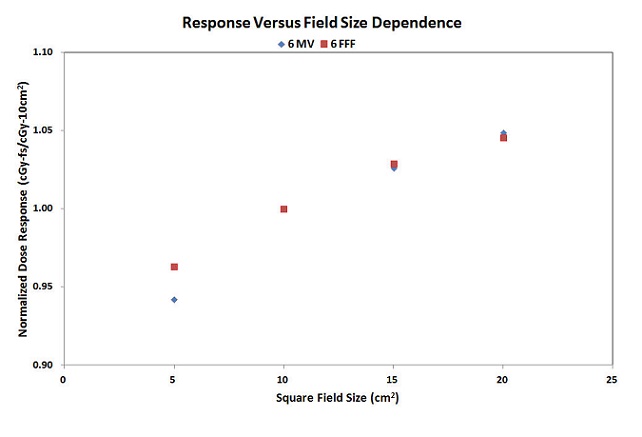

Supplement: Supplementary file 1 — Supplementary Material [file ACM2-15-212-s001.jpg]
